# Similarities in the Electrographic Patterns of Delayed Cerebral Infarction and Brain Death After Aneurysmal and Traumatic Subarachnoid Hemorrhage

**DOI:** 10.1007/s12975-024-01237-w

**Published:** 2024-02-23

**Authors:** Jens P. Dreier, Coline L. Lemale, Viktor Horst, Sebastian Major, Vasilis Kola, Karl Schoknecht, Michael Scheel, Jed A. Hartings, Peter Vajkoczy, Stefan Wolf, Johannes Woitzik, Nils Hecht

**Affiliations:** 1 Center for Stroke Research Berlin, Campus Charité Mitte, Charité – Universitätsmedizin Berlin, corporate member of Freie Universität Berlin, Humboldt-Universität zu Berlin, and Berlin Institute of Health, Charitéplatz 1, 10117 Berlin, Germany; 2 Department of Experimental Neurology, Charité – Universitätsmedizin Berlin, corporate member of Freie Universität Berlin, Humboldt-Universität zu Berlin, and Berlin Institute of Health, Berlin, Germany; 3 Department of Neurology, Charité – Universitätsmedizin Berlin, corporate member of Freie Universität Berlin, Humboldt-Universität zu Berlin, and Berlin Institute of Health, Berlin, Germany; 4 Bernstein Center for Computational Neuroscience Berlin, Berlin, Germany; 5 Einstein Center for Neurosciences Berlin, Berlin, Germany; 6 Institute of Neuropathology, Charité – Universitätsmedizin Berlin, corporate member of Freie Universität Berlin, Humboldt-Universität zu Berlin, and Berlin Institute of Health, Berlin, Germany; 7 Medical Faculty, Carl Ludwig Institute for Physiology, University of Leipzig, Leipzig, Germany; 8 Department of Neuroradiology, Charité – Universitätsmedizin Berlin, corporate member of Freie Universität Berlin, Humboldt-Universität zu Berlin, and Berlin Institute of Health, Berlin, Germany; 9 Department of Neurosurgery, University of Cincinnati College of Medicine, Cincinnati, OH USA; 10 Department of Neurosurgery, Charité – Universitätsmedizin Berlin, corporate member of Freie Universität Berlin, Humboldt-Universität zu Berlin, and Berlin Institute of Health, Berlin, Germany; 11 Department of Neurosurgery, Evangelisches Krankenhaus Oldenburg, University of Oldenburg, Oldenburg, Germany

**Keywords:** Stroke, Subarachnoid hemorrhage, Brain death, Electrocorticography, Neuromonitoring, Spreading depolarization

## Abstract

While subarachnoid hemorrhage is the second most common hemorrhagic stroke in epidemiologic studies, the recent DISCHARGE-1 trial has shown that in reality, three-quarters of focal brain damage after subarachnoid hemorrhage is ischemic. Two-fifths of these ischemic infarctions occur early and three-fifths are delayed. The vast majority are cortical infarcts whose pathomorphology corresponds to anemic infarcts. Therefore, we propose in this review that subarachnoid hemorrhage as an ischemic-hemorrhagic stroke is rather a third, separate entity in addition to purely ischemic or hemorrhagic strokes. Cumulative focal brain damage, determined by neuroimaging after the first 2 weeks, is the strongest known predictor of patient outcome half a year after the initial hemorrhage. Because of the unique ability to implant neuromonitoring probes at the brain surface before stroke onset and to perform longitudinal MRI scans before and after stroke, delayed cerebral ischemia is currently the stroke variant in humans whose pathophysiological details are by far the best characterized. Optoelectrodes located directly over newly developing delayed infarcts have shown that, as mechanistic correlates of infarct development, spreading depolarizations trigger (1) spreading ischemia, (2) severe hypoxia, (3) persistent activity depression, and (4) transition from clustered spreading depolarizations to a negative ultraslow potential. Furthermore, traumatic brain injury and subarachnoid hemorrhage are the second and third most common etiologies of brain death during continued systemic circulation. Here, we use examples to illustrate that although the pathophysiological cascades associated with brain death are global, they closely resemble the local cascades associated with the development of delayed cerebral infarcts.

## Introduction 

Subarachnoid hemorrhage (SAH) is a life-threatening cerebrovascular event. In about 85% of cases, it is caused by the rupture of a cerebral aneurysm. Damage of brain tissue in the context of aneurysmal SAH (aSAH) is due to (1) the initial global cerebral ischemia which at least in part might be due to increase in intracranial pressure (ICP), (2) the accumulation of blood in the subarachnoid space, (3) the intraventricular blood accumulation, (4) the direct destruction of brain tissue by intracerebral hemorrhage (ICH), and (5) the occurrence of focal cerebral ischemia. Focal ischemic damage develops both in the early phase (early cerebral ischemia (ECI)) (initial ~ 48 h) [1–3] and in the delayed phase (delayed cerebral ischemia (DCI)) [4–6] (48 h until ~ 2 weeks).

The prospective, observational, multicenter, diagnostic phase III trial DISCHARGE-1 recently found that cumulative focal brain damage from ICH, ECI, and DCI up to Day 14, as determined by neuroimaging, is the strongest known predictor of patient outcome half a year after the initial hemorrhage [7]. In DISCHARGE-1, on average 61.2 ± 100.3 ml (76.0%) of the total focal brain damage per patient was ischemic (ECI and DCI) and 19.4 ± 29.1 ml (24.0%) was hemorrhagic (ICH) (*p* < 0.001, *n* = 180, Mann–Whitney Rank Sum Test). In other words, about three-quarters of the focal brain damage after aSAH was ischemic. The average damage caused by ECI amounted to 26.9 ± 66.9 ml. The 170 survivors of the early phase developed an average volume of 36.3 ± 80.1 ml of damaged tissue due to DCI. The vast majority of these infarcts are located in the cerebral cortex and pathomorphologically they are almost without exception so-called anemic infarcts [3, 8–14].

Since severe aSAH is composed of different subtypes of stroke, in contrast to a spontaneous ICH [15], we believe it would be more accurate to refer to aSAH as an ischemic-hemorrhagic stroke rather than a simple hemorrhagic stroke. Thus, especially for research into the pathophysiological basis of the development of brain damage associated with cerebral ischemia in humans, the study of aSAH during neurocritical care is of great fundamental importance far beyond aSAH. For the following reasons, aSAH is even recognized as a model disease for cerebral ischemia in humans [16]: DCI usually occurs while the patient is already in the neurocritical care unit. Clinical studies on thrombolytic and endovascular treatment of ischemic stroke have shown that rescue therapies are most effective when administered early after the onset of ischemia [17, 18] summarized in the motto “time is brain” [19]. Patients with DCI could theoretically even be treated before the tissue damage begins. However, besides the fact that there is currently no effective, evidence-based therapy for DCI [20], another relevant difficulty is to recognize the onset of DCI in time, as typical patients with severe aSAH and high risk of DCI are often comatose. The latter is also the reason why it is so difficult to test novel approaches to the treatment of DCI in a meaningful way. However, to possibly overcome this difficulty, it is interesting that neurosurgical interventions are indicated in patients early after the initial hemorrhage so that invasive probes can be implanted. This provides a unique opportunity to invasively monitor the entire period of ischemic stroke development, perform early treatment stratification based on changes in diagnostic summary measures captured in real time via invasive neuromonitoring techniques, and then capture the responses of the diagnostic summary measures to the intervention to save the tissue. This corresponds to the concept of so-called precision (individualized) medicine [21]. Due to the recent refinement of minimally invasive procedures [22–24], this approach is currently becoming increasingly available also for patients who do not require craniotomy during neurocritical care.

In the last decade, major interim scientific successes on this road to precision medicine of DCI were human recordings, in patients with aSAH, that characterized the electrocorticographic (ECoG) hallmarks of (1) brain infarct development [25], (2) brain death development during continued systemic circulation [26, 27], and (3) brain death in the wake of cardiocirculatory arrest [28]. In addition, the dying process in the wake of cardiocirculatory arrest was also recorded in TBI patients and in one patient with malignant hemispheric stroke [28].

Severe TBI has become the other model disease for progressive brain damage during neurocritical care, as neurosurgical interventions allowing implantation of invasive probes are also often indicated after this condition [29–32]. In addition, bleeding into the subarachnoid space can also occur as a result of TBI, meaning that TBI patients are also at risk of DCI [33, 34]. Accordingly, there are important pathophysiological overlaps in the mechanisms between TBI and aSAH [22, 35], although DCI is overall more frequent and severe in aSAH patients [7] than in TBI patients [36, 37]. The further refinement of minimally invasive procedures [22–24] is also applicable to TBI, increasing the number of neurotrauma patients who might benefit from invasive neuromonitoring during neurocritical care.

Building on these introductory considerations, in the first part of this review, we will look at the epidemiological significance of aSAH as an ischemic-hemorrhagic stroke in comparison to purely ischemic and purely hemorrhagic (spontaneous ICH [15]) strokes. We will then briefly discuss the early phase and the delayed phase after aSAH and explain what spreading depolarizations (SD) are. On this basis, we will examine typical ECoG patterns of newly developing delayed cerebral infarcts, as intensivists will be increasingly confronted with such patterns due to the growing use of modern neuromonitoring techniques during the neurocritical care of aSAH and TBI patients. Finally, we will discuss typical ECoG patterns of brain death development during continued systemic circulation, as they occur globally but are otherwise very similar to patterns of local cerebral infarct development. In comatose patients in particular, we believe that understanding the nature and patterns of these harmful events and diagnosing them early, before it is too late to intervene, can make an important contribution to the development of therapeutic strategies to combat them. This is in line with the tenet espoused by Max Planck for science in general that “insight must precede application.”

## Epidemiological Relevance of aSAH

The estimated global lifetime risk of ischemic stroke from the age of 25 years onward is 18.3%, and the risk of hemorrhagic stroke is 8.2% [38]. Spontaneous ICH is the most common form of hemorrhagic stroke. Its incidence is estimated at 25 per 100,000 person-years [39]. Spontaneous ICH accounts for 10–20% of all strokes [40, 41]. The estimated median case fatality of ICH at 1 month is ~ 40% (range 13–61) [39]. In epidemiologic studies, SAH is usually considered the second most common form of hemorrhagic stroke, which, as explained above, is not consistent with our pathophysiologic view, in which SAH as an ischemic-hemorrhagic stroke is rather a third, separate entity. The incidence of SAH is estimated at 8 per 100,000 person-years [42, 43] and accounts for only 5% of all strokes [44]. Case fatality of SAH varies between 27 and 44% for individual regions [45, 46].

Although the overall incidence of SAH is lower than that of pure ischemic stroke or ICH, the relatively young average age of SAH patients means that SAH accounts for almost the same proportion of potential years of life lost to stroke before the age of 65 (YPLL, a measure of premature mortality). In a US study, for example, it was estimated that 38.5% of stroke-related YPLL were due to ischemic stroke, 34.2% to ICH, and 27.3% to SAH (cf. Table 2 in Johnston et al. [47]). The estimated mean age of occurrence is 55 years for SAH [20], 65 years for ICH [48], and 70 years for ischemic stroke [49]. The estimated median age of death from SAH is 59 years compared with 73 years for ICH and 81 years for ischemic stroke [47]. The incidence of SAH in women is globally estimated to be 1.2 (95% confidence interval (95% CI), 1.1–1.4) times higher than in men [44]. In DISCHARGE-1, the median age was 55 years (interquartile range (IQR), 47–63) and the female-to-male ratio was 2.1:1 [7], which corresponds well with the nationwide female-to-male ratio in Germany of 1.8:1 [43]. The sex ratio is reverse for spontaneous ICH [48]. Thus, for example, in US women of European genetic background, an estimated 35.8% of stroke-related YPLL are due to SAH, while ischemic strokes and ICH account for only 34.7% and 29.5%, respectively [47].

Based on 1794 patients from four randomized clinical trials of tirilazad, sex was no predictor of poor patient outcome after aSAH [50]. However, not only are women more frequently affected by aSAH, but the negative socio-economic effects of aSAH also appear to be more serious for women than for men in a direct comparison. For example, in a study from Sweden, having sickness absence and/or disability pension at the end of the 3-year follow-up was predicted by a model consisting of (1) female sex, (2) living in a village/rural area, and (3) having a defined bleeding source for the SAH [51]. The female dominance in SAH has yet another unfavorable side effect, because in almost three-quarters of cases where a disease predominantly affects one sex, science funding is biased towards men, i.e., either the disease affects more women and is underfunded (in terms of burden) or the disease affects more men and is overfunded [52].

Although this is poorly captured in epidemiological studies [53], case fatality rates are likely to vary widely around the world since treatments for aSAH, which have led to better outcomes in countries with higher income [45], are among the most expensive in the entire healthcare system. This is because patients with aSAH are usually treated in critical care units according to the severity of the disease. In typical high-income countries such as Germany, for example, critical care accounts for around 20% of hospital costs with a share of only around 5% of hospital beds [54]. In addition, SAH is one of the four conditions that are most strongly associated with high costs among all critical care conditions [55]. The other three major independent cost drivers in critical care are (1) younger age, (2) acute respiratory failure requiring ventilation, and (3) complications related to procedures. There are only a few estimates of the total economic burden of SAH. For example, in 2005, this burden was estimated at £510 million annually in the UK alone [56]. This figure is related solely to SAH due to the rupture of a cerebral aneurysm.

The overall value of neurocritical care and emergency medicine is supported by a decrease in aSAH case-fatality rates by 17% between 1973 and 2002 [45]. In addition, the proportion of patients who regained independent function increased [53]. However, the curve of outcome improvement has leveled off over time and has recently stagnated. In particular, the prevalence of DCI did not decrease over time, as recently estimated by means of a random-effects meta-analysis of randomized controlled trials [57]. This stagnation is reflected in the fact that SAH patients remain the third most common group to experience brain death prior to circulatory collapse during critical care, following ICH and TBI in the top ranks. SAH is therefore also the third most prevalent disease as a source for organ transplantation [58].

## Early and Delayed Phase After aSAH

In their review, Macdonald and Schweizer defined the delayed phase of brain injury by DCI as the period 3–14 days after the initial hemorrhage [20]. The Co-Operative Studies on Brain Injury Depolarizations (COSBID) group (www.cosbid.org) uses a naming convention, following the practice in most countries, of counting the first 24-h period after the initial insult as “Day 0,” the next 24-h period as “Day 1,” and so on [59]. Accordingly, “Day 2” in the COSBID studies including the recent DISCHARGE-1 trial on aSAH corresponds to the third day in the review by Macdonald and Schweizer, and the DCI phase would generally begin 48 h after the initial injury.

Based on the neuromonitoring data, however, there is overall a rather continuous transition from the early to the delayed phase with a statistical decrease in the initial damage processes up to Day 3 followed by a renewed increase from Day 4 (see supplementary Fig. 3 in Dreier et al. [7]). Accordingly, demarcated ischemic events in neuromonitoring can occur not only in the delayed phase, but also on any early day after the initial hemorrhage [25]. As a rule, this is hardly noticeable clinically, as most patients at risk of these events in the early period are deeply comatose. The classification into an early and a delayed phase after aSAH is statistically justified and helps clinicians and clinician scientists to simplify orientation. Nevertheless, it is important not to interpret this rigidly, but to bear in mind that biologically there is rather no precisely definable temporal boundary between the two phases.

**Fig. 1:**
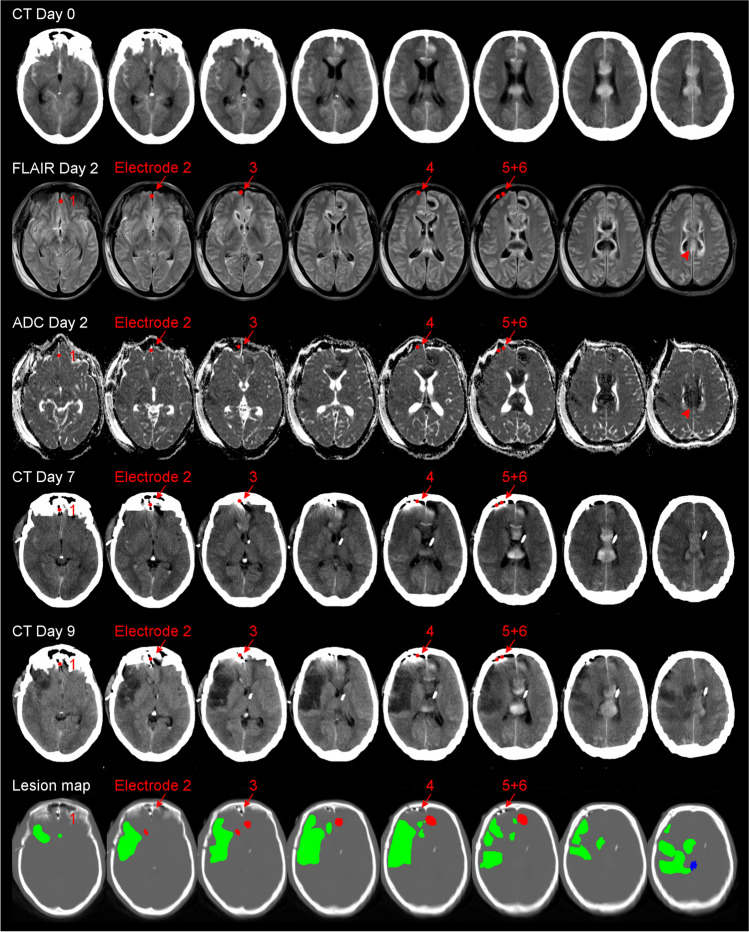
Development of a large delayed ischemic infarct between an MRI scan on Day 2 and two CT scans on Days 7 and 9 in a 46-year-old woman with aneurysmal subarachnoid hemorrhage (aSAH) due to rupture of an anterior communicating artery aneurysm. Representative CT and MRI slices in axial orientation. All images were aligned to the first postoperative T1 spin echo scan with a slice thickness of 5 mm. The initial CT scan on admission demonstrated subarachnoid blood in the basal cisterns and more superior in the callosal cistern and the interhemispheric fissure. Blood from the callosal cistern extended into the right frontal lobe and formed a small intracerebral hemorrhage (ICH) of 1 ml at a distance of 24 mm from subdural electrode 1. A small ICH with a volume of 4 ml was also found in the left medial frontal lobe originating from subarachnoid blood in the left cingulate sulcus. The postoperative MRI scan 52 h (Day 2) after onset of the initial hemorrhage showed symmetric, hyperintense bands in the cortex of the left and right superomedial margin on fluid attenuation inversion recovery (FLAIR) images with restricted diffusion on the apparent diffusion coefficient (ADC) map (see arrowheads). Furthermore, four hyperintense spots were found on diffusion-weighted imaging (DWI) in the right frontal and occipital cortex, the left frontal cortex, and the cerebellum (not shown). These findings are consistent with early brain infarction. Quantification yielded an infarct volume of 3 ml in the right hemisphere (shortest distance between infarct border and subdural electrode 5, 29 mm), 2 ml in the left hemisphere, and 1 ml in the cerebellum. Also note perihematomal edema surrounding ICH in both the right and left frontal lobe (hyperintense on FLAIR images, hypointense on the ADC map). On Day 7, CT revealed loss of gray-white matter differentiation in the right insular and frontal cortex. On Day 9, hypodensity and swelling became more marked with a midline shift of 6 mm and compression of the lateral ventricles. These findings are consistent with delayed cerebral infarction involving the vascular territories of the right middle cerebral artery (MCA) and anterior cerebral artery (ACA). Volume analysis revealed a tissue loss of 107 ml due to delayed cerebral ischemia (DCI) ipsilateral to the subdural electrodes. The lowest row presents the CT scan (bone window) on Day 9. The subdural electrode strip is visible over the medial and lateral surface of the right frontal lobe (electrodes are marked with red arrows). Additionally, the quantified lesions from the scans above are projected onto the CT images. The red-labeled area indicates ICH, the blue area early infarction in the MRI on Day 2, and the green area delayed infarction in the CT on Day 9. Note the proximity between the delayed ischemic infarct and electrode 6, which was at 6 mm from the infarct

**Fig. 2:**
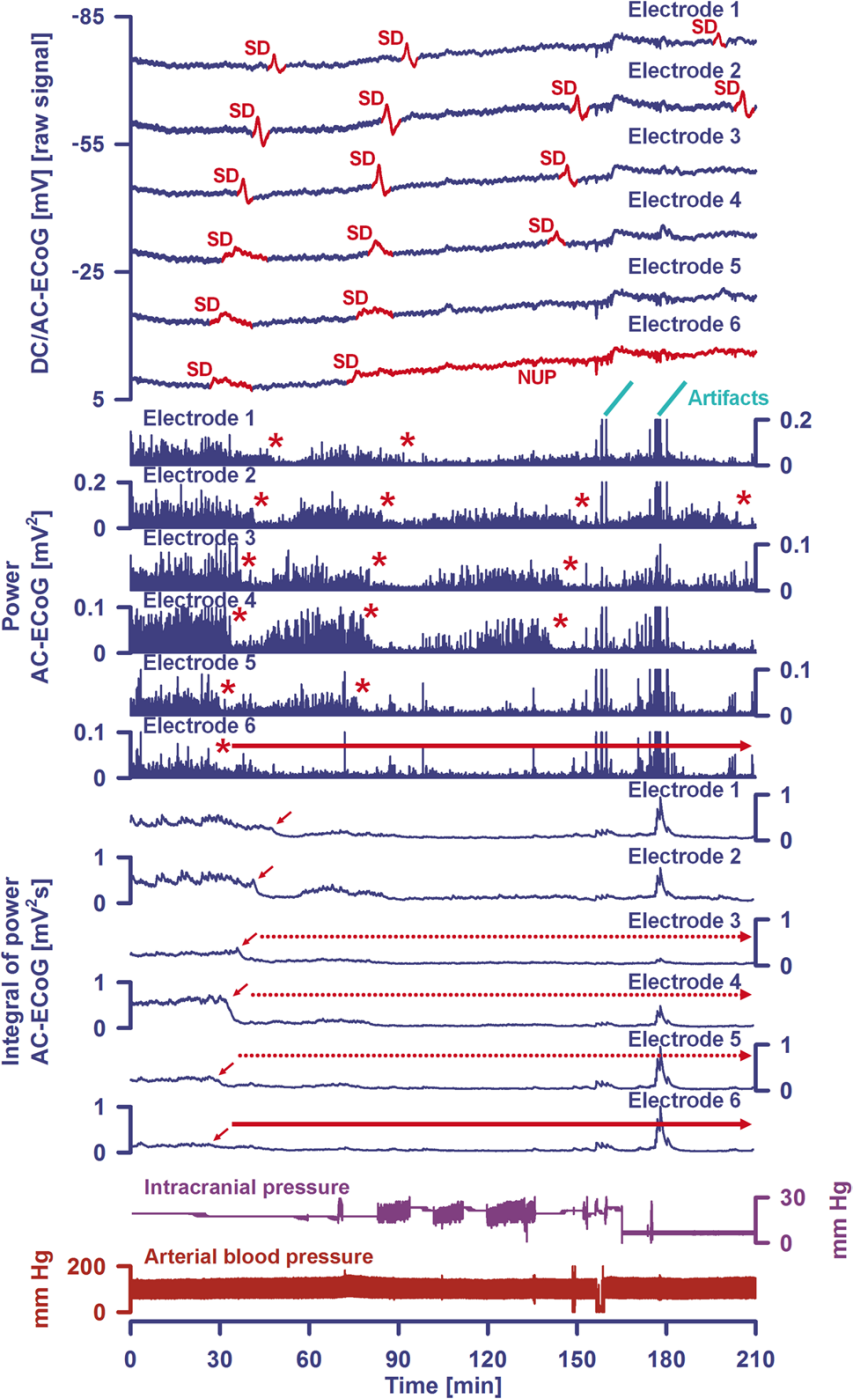
A cluster of spreading depolarizations (SD) precedes the development of the large delayed ischemic infarct that is shown in Fig. 1 between the MRI scan on Day 2 and the CT scan on Day 7. The patient already had 6 single SDs before the SD cluster shown started on Day 6. Five of those also occurred on Day 6. Traces 1–5 give the raw direct current (DC)/alternating current (AC) electrocorticography (ECoG) recordings (bandpass: 0-45 Hz), demonstrating the propagation of the negative DC shifts along the cortex from electrode 6 to electrode 2, which identify the SDs. The ECoG traces are oriented according to the convention of electroencephalography (EEG) with negativity up and positivity down. The distance between two neighboring electrodes is always 1 cm. As shown in Fig. 1, electrode 6 was located at 6 mm from the infarct which was first seen as a loss of gray-white matter differentiation on the CT scan of Day 7 5 h after onset of the SD cluster. Note that the negative DC shifts that were closer to the developing infarct were longer than those that were further away from the developing infarct. At electrode 6, the DC signal no longer returned to the baseline, i.e., a shallow negative ultraslow potential (NUP) emerged there. In contrast, NUPs measured in the core of newly developing infarcts have a much greater amplitude [25]. The depressive effect of the SDs on the spontaneous activity is assessed here using the power (traces 7–12) and the integral of the power (traces 13–18) in the AC frequency band between 0.5 and 45 Hz (red asterisks and short red arrows mark the onset of spreading depression). It is relevant to first identify the artifacts and not to include them in the assessment of the durations of the depression periods. Importantly, the spontaneous activity remained persistently depressed at electrode 6 (red arrow), resulting in a very long peak total SD-induced depression duration of a recording day (PTDDD). PTDDD of the delayed neuromonitoring period (PTDDD_delayed_) is the strongest currently known real-time predictor of delayed cerebral ischemia (DCI) [7]. If the recording strip had been placed further forward and only electrodes 3–5 had been available to determine the duration of the longest depression period, the evaluation would have been more difficult, as the power of spontaneous activity at these electrodes showed some recovery relatively quickly. However, the integral of power demonstrates that the recovery of spontaneous activity in these electrodes was indeed minimal (red dotted arrows). The intracranial pressure (ICP) was measured via an external ventricular drain (trace 19) and the arterial pressure via a catheter in the radial artery (trace 20)

**Fig. 3:**
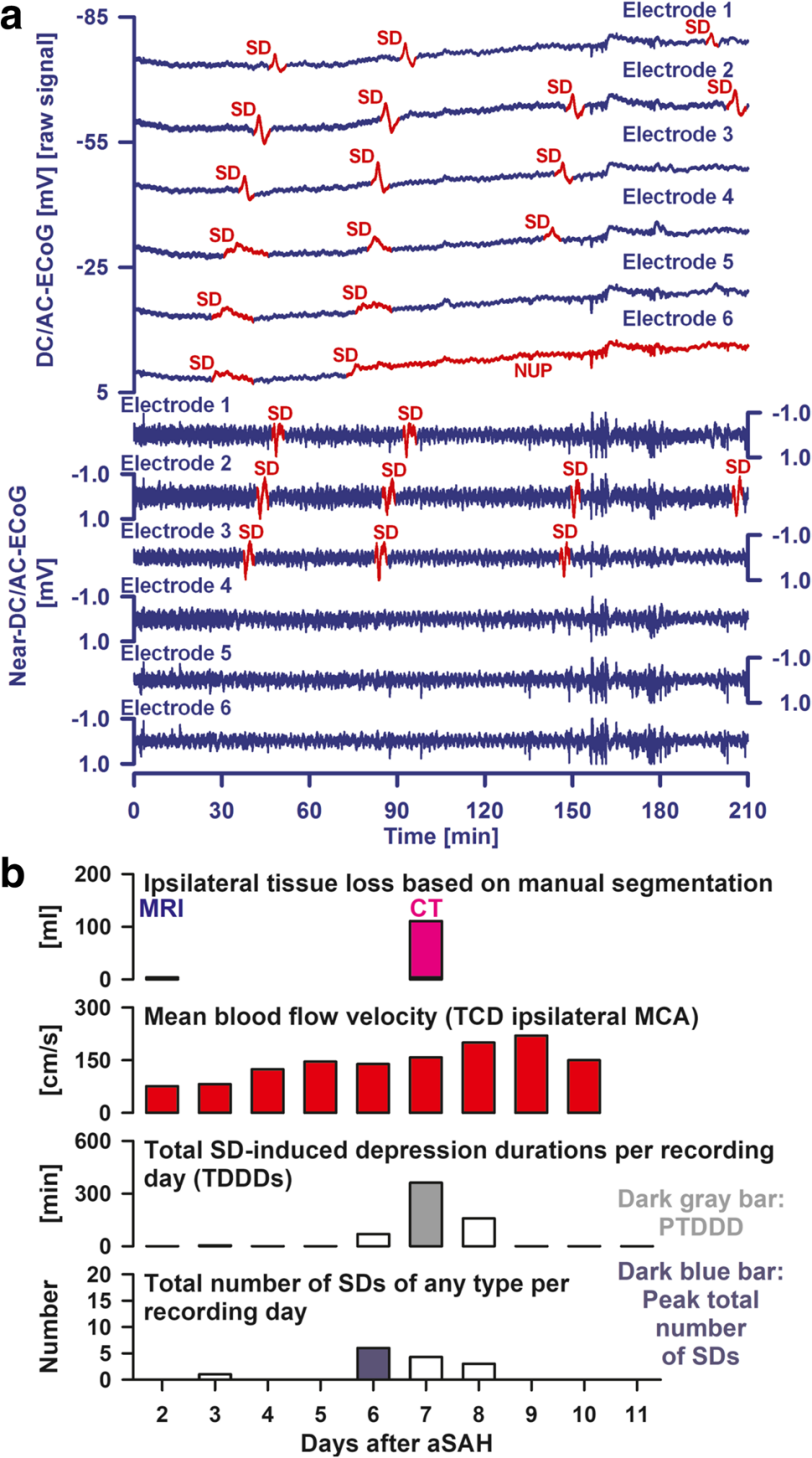
**a** A direct current (DC)/alternating current (AC)-electrocorticography (ECoG) amplifier is superior to a near-DC/AC-ECoG amplifier for recording spreading depolarizations (SD) and the development of cerebral infarction. Traces 1–6 show the same raw DC/AC-ECoG recordings (band-pass, 0–45 Hz) as traces 1–6 in Fig. 2. However, traces 7–12 give the near-DC/AC-ECoG recordings (band-pass, 0.01–45 Hz) to illustrate that the longer negative DC shifts at electrodes 4–6 near the developing infarct become invisible in the near-DC/AC-ECoG recordings. In other words, the occurrence of SDs can be paradoxically underestimated in regions where SD is most important, if only near-DC/AC-ECoG recordings are available. The ECoG traces are oriented with negativity upward and positivity downward according to the electroencephalography (EEG) convention. **b** Row 1 from top to bottom shows the progression of focal brain damage from the MRI on Day 2 to the CT on Day 7, when the delayed infarct first became visible in the form of a loss of differentiation between gray and white matter in the right insular and frontal cortex (see Fig. 1). Row 2 shows the time course of the transcranial Doppler-sonography (TCD)-measured mean blood flow velocity in the ipsilateral middle cerebral artery (MCA). Rows 3 and 4 show the time course of the SD variables: For each day, SDs were counted and depression durations were scored to determine the total duration of SD-induced activity depression per recording day (TDDD) (row 3) and the total number of SDs per recording day (row 4). The peak TDDD (PTDDD) and peak SDs/day were defined for each patient as the maximal values among all recording days (indicated as a dark gray and dark blue bar, respectively). The delayed SDs started on Day 6 and reached its maximum on Day 7, i.e., they started many hours before the early signs of delayed infarction were seen on CT (compare CT on Day 7 in Fig. 1). The highest TCD-measured mean blood flow velocity in the ipsilateral MCA was 220 cm/s measured on Day 9 and the ipsilateral qualitative digital subtraction angiography (DSA) score was 2.57 on Day 9 suggesting that the patient had severe angiographic vasospasm on the affected side. However, on Day 6, when the electrographic processes leading to infarction began, the TCD-measured mean blood flow velocity was only 139 cm/s. On Day 7, when the delayed infarction was already visible on CT by the loss of differentiation between gray and white matter, it was only 158 cm/s. This temporal lag of the TCD-measured peak mean blood flow velocities after infarct development was typical for DISCHARGE-1 (compare Fig. 3Cv in Dreier et al. [7]), if the delayed infarcts were accompanied by proximal vasospasm at all (compare Fig. 7E in Dreier et al. [7])

As an example of the development of focal brain damage after aSAH, Fig. 1 shows an ICH and damage from ECI on postoperative MRI on Day 2 and the development of a large delayed ischemic infarct between the early MRI scan on Day 2 and two CT scans on Days 7 and 9 in a typical female patient in her forties. In order to ease understanding of the electrographic basis of the delayed infarct development in this patient, which is explained in Figs. 2 and 3, we would like to provide some general background information on SDs in the following.

## The Role of SD in the Pathogenesis of Acute Brain Injury

Although SD is also presumably the mechanism of the comparatively harmless migraine aura [60–63], SD is primarily and with few exceptions [64] the key mechanism to understand how ischemic damage in gray matter of the nervous system occurs in virtually all species with a circulatory system [16, 65–67]. It is relatively irrelevant in this regard whether the ischemia occurs globally, e.g., in the context of circulatory arrest [28, 68], or focally, e.g., due to occlusion of cerebral arteries [69]. Whoever is interested in understanding this process may, for example, watch video 1 by Zhao et al. [69]. This is an illustrative example of how SD in focal cerebral ischemia develops in a single point in the still completely vital tissue in the ischemic core and spreads concentrically from there over the entire hemisphere. The depolarized state induced by this initial SD wave then persists in the core, where it progresses to infarction, while the depolarization recovers and regresses in the non-ischemic cortex. Unfortunately, there is a persistent myth that ischemic infarction in the cortex can develop independent of SD, but this is an outdated understanding and, in view of advances in recent decades, there is no experimental or clinical evidence to support it.

The first scientific evidence that SD comes first and death follows dates back to a 1947 study by Aristides Leão himself [66], who discovered the SD phenomenon in 1944 [70]. The sequence he described has been reproduced many times since then [25, 71] and has been observed in all properly studied animals, from insects to fish and further upwards in phylogenesis. It has now also been confirmed in humans during circulatory arrest, during the development of brain death with continued systemic circulation, and during the development of focal cerebral infarction [25–28]. The only in vivo exception to the sequence is found under extreme experimental manipulation, when astrocytic function is severely compromised prior to onset of severe ischemia. In this case, the typical sequence of SD followed by cell death is undermined, and cell death may occur in the ischemic core more or less simultaneously with a non-spreading neuronal and astrocytic depolarization [72]. Some may doubt the causal necessity of SD for cell death in vivo on the grounds that neurons die under hypoxia in cell cultures that lack the conditions for SD development. However, in these non-physiologic conditions, neuronal cell death only develops after many hours longer than in vivo [73].

SD is characterized by the near-complete breakdown of the transmembrane ion gradients, cytotoxic edema, and the release of numerous neurotransmitters, including glutamate [74–76]. From an electrochemical and thermodynamic perspective, SD refers to the abrupt transition from the physiological double Gibbs-Donnan steady state between intracellular and extracellular space, characterized by low entropy, to a state close to a stable Gibbs-Donnan equilibrium characterized by high entropy [77, 78]. SD is thus the most fundamental electrochemical change that the living brain can experience and represents a disruption in homeostasis that is orders of magnitude greater than that observed, for example, during an epileptic seizure [79–82]. For a more comprehensive account of the thermodynamic principles of SD, we would like to refer the reader to the following review [75].

SD is measured as a large negative direct current (DC) shift, which can be recorded in humans using a subdural electrode strip and indicates the depolarized state of tissue [59] (Fig. 2). In typical electroencephalography frequency bands (AC-ECoG), SD classically triggers a rapidly developing reduction in the amplitudes of spontaneous activity, known as spreading depression [70] (Fig. 2). However, the changes in neuronal activity that may be associated with SD, even when SD spreads in metabolically intact tissue, are often more complex than just spreading depression [59, 82–84]. Measured at the surface of the brain, SD can even lead to an increase in normal brain activity, the so-called “boom” [83]. SD typically propagates in the tissue at a speed between 2 and 9 mm/min [85–87]. In certain conditions, however, speeds of up to 15 mm/min have been recorded [88]. For the question of neuronal death or survival during ischemia, the intraneuronal compartment is evidently the most decisive among all cerebral compartments. The term SD describes the abrupt cytotoxic change that leads to edema [74, 89], a more than 100-fold increase in cytoplasmic calcium concentration [67, 90], a three-fold increase in cytoplasmic sodium concentration [91], and a host of other changes in the neuronal cytoplasm that are beyond the scope of this review. Both calcium and sodium are important second messengers [92–94]. It is assumed that the extreme deflections of these second messengers alone during SD are highly toxic if they last too long [79, 95].

**Fig. 4:**
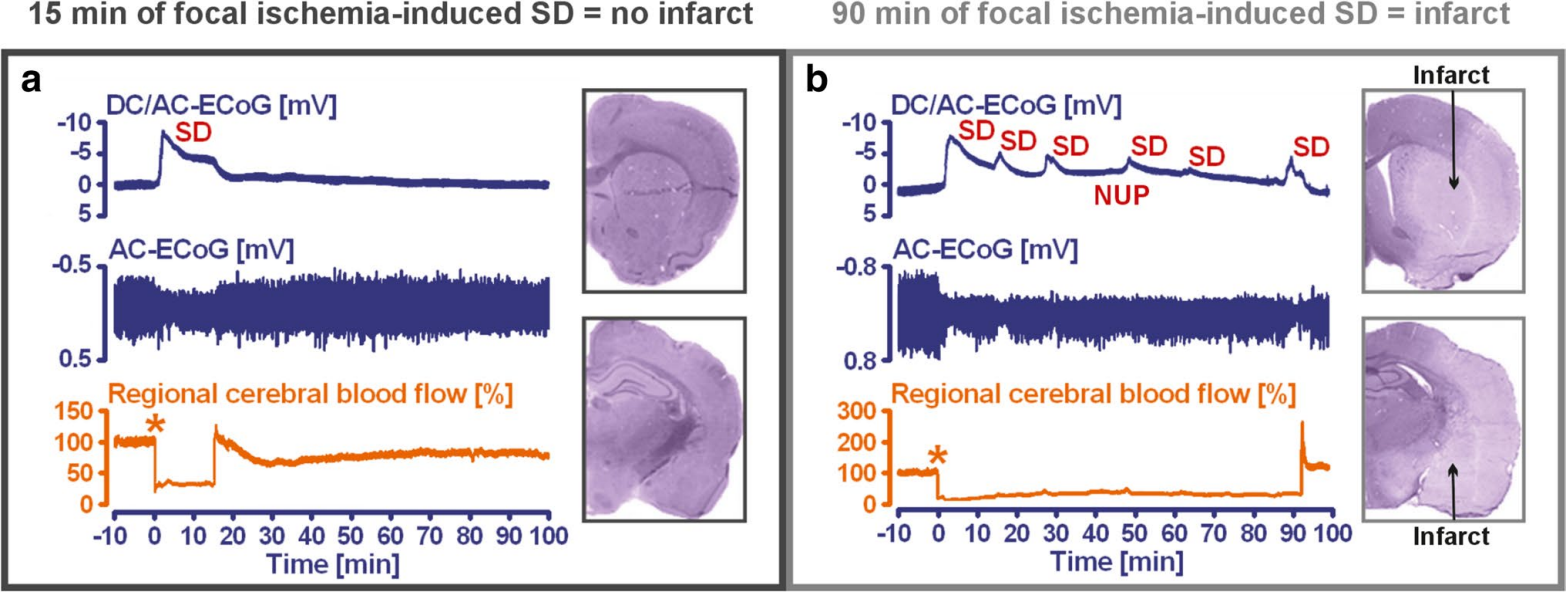
Two exemplary original experiments on Sprague–Dawley rats from the study by Lückl et al. are shown here [25]. After head surgery and placement of a laser-Doppler flowmetry (LDF) probe to measure regional cerebral blood flow (rCBF) (trace 3 from top to bottom) and electrodes to measure direct current (DC) (trace 1)/alternating current (AC) (trace 2)-electrocorticography (ECoG), the filament was later advanced during the experiment until the LDF probe indicated adequate middle cerebral artery occlusion (MCAO) by a sharp decrease in rCBF. The ECoG traces are oriented with negativity upward and positivity downward according to the electroencephalography (EEG) convention. As can be seen in trace 2, focal cerebral ischemia triggers a rapidly developing reduction in the amplitudes of spontaneous brain activity within a few seconds, which typically begins practically simultaneously in the entire ischemic region (= non-spreading depression of activity) [7, 16, 25]. The initial spreading depolarization (SD), which can be observed as a negative DC shift in trace 1, only began after the onset of the non-spreading depression with a time delay. After either 15 min (**a**) or 90 min (**b**) of occlusion, the filament was gently withdrawn and the reperfusion was monitored. After 72 h of survival, the animals underwent cardiac perfusion fixation and histological assessment. Infarcts are macroscopically identified as pale areas using hematoxylin staining (cf. right hemisphere in (**b**)). When the animals were sacrificed 72 h after transient MCAO, no dead neurons were found in the ischemic core if the ischemic core was reperfused within 15 min, even though perfusion was very low and neurons were persistently in the state of SD during the ischemic period (**a**). In contrast, 90 min of MCAO and ischemia-induced SDs superimposed on a negative ultraslow potential (NUP) typically resulted in a large cerebral infarct (**b**). Such experiments prove that SD is an initially reversible process. The neurons only die if the depolarized state known as SD persists beyond a threshold duration, the so-called commitment point [95]. In this context, it is important to understand that the commitment point is not a universal value but is influenced by other factors such as perfusion level, temperature, and type of neuron [75, 107, 173]

Typically, the first SD starts in the ischemic core 2 to 5 min after the onset of ischemia [66, 68, 96–99]. However, depending on the severity of ischemia, latencies of more than 30 min between the onset of ischemia and the first SD have also been described [69]. Importantly, SD does not mark the beginning of cell death but starts the countdown to cell death [71, 79, 100]. If the depolarized state induced by SD persists beyond a threshold duration, the so-called commitment point, the neurons die [95]. This implies that neurons can survive SD in the ischemic core if the tissue is reperfused and repolarizes before the commitment point [25, 71, 101–106]. For example, after MCAo in rats, at least 15 min must elapse during which neurons are in the state of SD before the first neurons in the ischemic core begin to die. This means that when the animals are sacrificed 72 h later, no dead neurons are found in the ischemic core if the ischemic core was reperfused within 15 min, even though perfusion was very low and neurons were persistently in the state of SD during the ischemic period (Fig. 4). Generally, the lower the residual blood flow in the ischemic tissue, the shorter is the time until the commitment point is reached [107]. The mechanism of cell death is predominantly necrosis when neurons experience very long-lasting SD of around 30–60 min or longer. This shifts towards apoptosis and, hence, to slower death within the necrotic-apoptotic continuum when there is local reperfusion and recovery from SD after the commitment point but before ~ 30–60 min [102, 108].

Repolarization after SD (reversal of the DC shift) requires activation of energy-dependent membrane pumps such as Na,K-ATPases [79, 109]. Short-lasting DC shifts thus indicate enough ATP at the recording site to fuel repolarization. This feature makes the negative DC shift duration a useful measure for (1) the tissue energy status and (2) the risk of injury at the recording site [59]. Terminal SD is roughly characterized by the initial reversible SD phase followed by the phase of the negative ultraslow potential (NUP) associated with irreversible functional damage [3, 16, 25, 110–112]. Terminal SD is observed, for example, when the brain dies as a whole [7, 26–28], when the core of a focal ischemic zone dies [25, 112], or when penumbral tissue dies in the further course of infarct maturation [113, 114]. One limitation is that the commitment point can be roughly estimated from the ECoG changes but cannot be precisely determined on ECoG criteria alone. Overall, it is surprisingly difficult to determine the exact point in time at which a loss of function becomes irreversible, even for a single neuron.

The concept of the SD continuum is crucial for clinical neuromonitoring because many SDs observed in patients have intermediate features, as opposed to the two extremes of SD in either severely ischemic or normal tissue [28, 79]. This is relevant not only for the correct bedside reading of ECoGs but also because there are major differences in mechanistic aspects and pharmacological sensitivity of SDs along the continuum. These differences are dictated by local tissue conditions and have implications for the efficacy of therapeutic targeting. Furthermore, although SD is a near-complete breakdown of homeostasis with toxic changes that typically precede ischemic cell death, it is possible that SD could also be adaptive in regions where there is sufficient energy in the tissue to survive it [16, 115]. In this context, it is, for example, interesting that optogenetically triggered SDs outside the ischemic zone in mice did not cause additional ischemic damage [116], in contrast to KCl-triggered SDs outside the ischemic zone in rats [117–119]. The optogenetic triggering of SDs is obviously gentler on the tissue than triggering with KCl [79]. A limitation of this murine study is that no direct measurements of SDs were performed, i.e., SDs were neither recorded electrophysiologically nor visualized with, for example, voltage-sensitive dye or calcium imaging, but the rCBF curves shown in Fig. 4 of this study suggest that in the mouse model chosen, only short-lasting SDs were optogenetically triggered [116]. These short-duration SDs did not appear to cause additional damage, which is in good agreement with the above statement that SDs must last longer than 15 min at a given location before irreversible local damage occurs there [25]. Importantly, in contrast to the mouse model of Sugimoto et al. [116], SD clusters with a spontaneous transition from short-duration to long-duration SDs can occur in aSAH patients and, e.g., in MCAO models in rats, typically leading to tissue death at the site of long-duration SDs (for example, compare Fig. 6 in [25], Fig. 7 in [112], Fig. 5 in [111], and Fig. 2 in [113]). This leads to two conclusions: (1) it is important to consider the local duration of SDs to make statements about local progression of damage, while it is not sufficient to simply count SDs [59, 120], and (2) there are conditions under which SDs are harmless [121] or perhaps even beneficial [16, 115]. In this regard, it is interesting to note that SD-like waves occur also in insects and worms [65, 122], and perhaps even in plants, where they are interpreted as a defense mechanism, e.g., when a caterpillar bites into a leaf and the wave spreads over the entire plant [123]. Based on such analogies, SD could perhaps be interpreted as an orderly retreat, which is not a favorable sign, but better than a chaotic collapse of the densely packed cells in most gray matter structures.

**Fig. 5:**
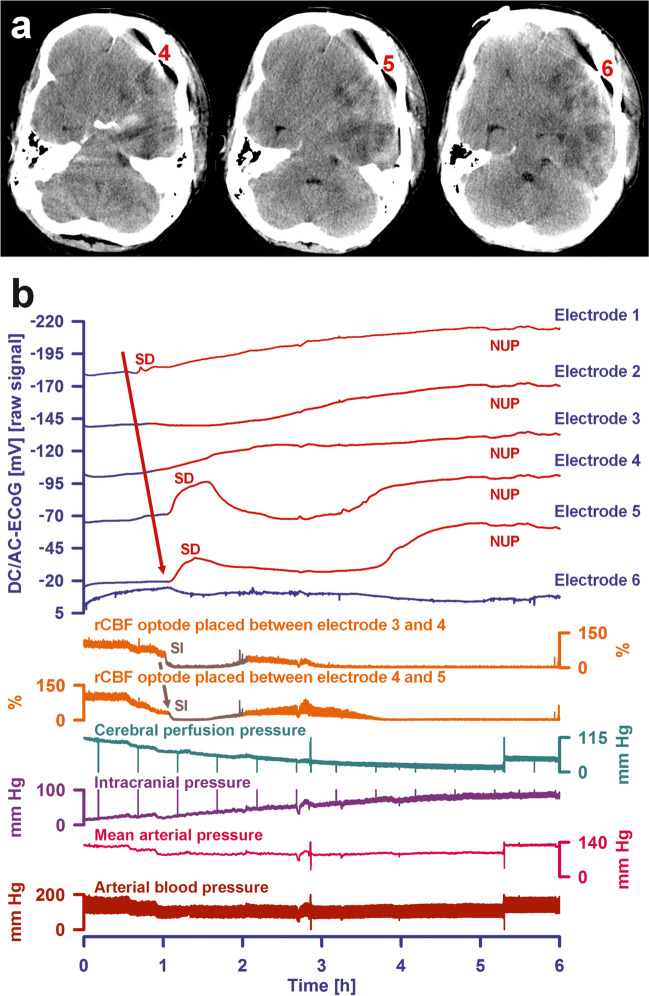
Spreading depolarization (SD) in a 44-year-old patient with aneurysmal subarachnoid hemorrhage (aSAH) that induced a spreading ischemia (SI) and preceded a terminal crisis of intracranial pressure (ICP), resulting in brain death. **a** The patient developed a large, ipsilateral delayed infarct with a volume of 107 ml between two CT scans on Days 8 and 12 [25]. The three slices of the CT from Day 12 show the scattered infarct with both hypo- and normodense parts below the three electrodes labeled “4,” “5,” and “6.” Electrodes 4 and 5 were located over the periphery and electrode 6 over the center of the scattered infarct. **b** Traces 1–6 from top to bottom give the direct current (DC)/alternating current (AC)-electrocorticography (ECoG) recordings (band-pass, 0–45 Hz) where SDs are recorded as large negative DC shifts. The ECoG traces are oriented with negativity upward and positivity downward according to the electroencephalography (EEG) convention. The following two curves (7 and 8) show the regional cerebral blood flow (rCBF) recorded with two optodes for laser Doppler flowmetry located between electrodes 3 and 4 and 4 and 5, respectively. Traces 9–12 give (1) the cerebral perfusion pressure (= mean arterial pressure (MAP)–ICP), (2) ICP measured with an external ventricular drain, (3) MAP, and (4) arterial pressure measured with a catheter in the radial artery. The last SD is shown that occurred on Day 12. It started at electrode 1 outside of the infarcted area from where it spread to all other electrodes apart from electrode 6. At electrodes 1–5, the last SD then transformed into a negative ultraslow potential (NUP) with an average amplitude of − 35 mV. The two optodes showed the typical pattern of SD-induced spreading ischemia that propagated from optode 3/4 to optode 4/5 and lasted for approximately 1 h. Afterwards, there was a transient partial recovery of rCBF at both optodes and a simultaneous partial recovery of the DC negativity at electrodes 4 and 5. However, ICP showed a dramatic increase in the wake of this last SD. Starting from a level of approximately 15 mmHg, it slowly increased to a level of approximately 60 mmHg, while MAP remained stable at 95 mmHg. Accordingly, cerebral perfusion pressure fell below 40 mmHg. In parallel, after the brief period of partial recovery, rCBF fell again at both optodes and the DC potential at electrodes 4 and 5 showed another swing towards negativity. Shortly after these events, ICP exceeded 90 mmHg, cerebral perfusion pressure fell further to 15 mmHg, and the patient was found to have loss of brainstem reflexes and fixed dilated pupils. After a discussion with the family regarding the poor prognosis, the patient was terminally extubated and expired

However, in mammals, in addition to the initial energy state at the beginning of the wave, neurovascular coupling also plays a key role in determining whether SD is locally harmless or harmful [16]. Thus, in otherwise normal tissue, SD acts as a potent stimulus to increase regional cerebral blood flow (rCBF) (= spreading hyperemia). After repolarization, this increase is typically succeeded by a physiological, prolonged, moderate hypoperfusion (= oligemia) [124, 125]. Under these conditions, neurons survive SD [121]. However, when neurovascular coupling is disturbed, SD can cause severe vasoconstriction instead of vasodilation, resulting in a long-lasting local perfusion deficit that prevents tissue repolarization and can eventually even lead to large cortical infarcts [126, 127]. The electrographic hallmark of this process is the prolonged negative DC shift [126] (Fig. 5). In short, this means that while SD is the usual consequence of cerebral ischemia, it can also be causal in inducing or worsening ischemia [16].

The phenomenon of SD-induced spreading ischemia was originally discovered in a rat model mimicking the conditions after aSAH [126]. On this basis, the first clinical study to detect SDs after aSAH in 2006 provided preliminary evidence that delayed neurological deficits (DND) are associated with a cluster of SDs [5]. The occurrence of SD-induced spreading ischemia was first demonstrated in aSAH patients in 2009 [22]. A translational study from 2017 then showed that focal accumulation of subarachnoid blood is sufficient to trigger SD clusters and early infarcts in a porcine model, and that phenomenologically, nearly identical early neuromonitoring and neuroimaging findings also occur in aSAH patients [3]. Using neuromonitoring technology in combination with longitudinal neuroimaging, the entire sequence of early and delayed brain infarct development following aSAH was shown in 2018 in a small patient population. In sum, recordings from optoelectrodes located directly over newly developing infarcts demonstrated SD-induced persistent activity depression, SD-induced spreading ischemia, and the transition from clustered SDs to NUP [25]. In the same year, the first clinical drug trial targeting SD-induced spreading ischemia was published [128]. The study investigated the endothelial nitric oxide synthase (NOS) activator cilostazol and showed a trend towards less DCI in the treatment group. Accordingly, the total duration of SD-induced depression per recording day was significantly lower in the cilostazol group. These associations were later confirmed in 2022 when the DISCHARGE-1 study found that SD variables contributed to every multiple regression model of early, delayed, and total brain damage after aSAH (and also patient outcome and death), strongly suggesting that they are an independent biomarker of progressive brain injury [7]. SDs were not only significantly associated with the evolution of delayed brain infarcts in this study, but also with reversible DNDs, which in clinical parlance are most closely equated with transient ischemic attacks (TIA). In contrast, the typical symptoms of a migraine aura in connection with an ECoG-recorded SD were actually only noticed in one aSAH patient in a total of over 200 patients with several thousand SDs [60]. In this one patient, however, there was a clear temporal connection with SD in two consecutive aura episodes.

In 2023, Horst et al. [10] then demonstrated that SD variables are a statistical mediator between subarachnoid blood volume and delayed infarct volume, while angiographic vasospasm is a mediator between intraventricular blood volume and delayed infarct volume in aSAH patients. However, no correlation was found between SD variables and angiographic vasospasm. Although there are hypotheses [3, 16, 129], the exact mechanism by which many SDs develop in patients with severe aSAH remains a mystery. The cause of SDs could well be multifactorial. However, we consider it likely that there is a principal pathogenesis axis in the development of cortical infarcts after aSAH [3, 8–14] that originates from toxic subarachnoid blood products [20, 130–132] and leads to SD-induced spreading ischemia via (1) mechanisms of SD initiation and (2) altered vasoreactivity in the cortical microcirculation [16, 126], with (3) mechanisms of local neuroinflammation probably also involved [133, 134]. SD-induced spreading ischemia would then in turn lead to the development of infarction if it causes the depolarized state to persist beyond the commitment point. In our opinion, proximal vasospasm is not a necessary prerequisite for the development of this pathophysiologic cascade (Fig. 3b) [4, 7, 135–139]. However, the statistical results in the DISCHARGE-1 population [7, 10] and animal data [140, 141] suggest that proximal vasospasm may enhance the processes in the neurovascular unit that lead to the development of cortical infarcts after aSAH.

## Neuromonitoring-Based Detection of DCI

In DISCHARGE-1, the best real-time predictor of DCI in the hemisphere equipped with the subdural electrode strip was the peak total SD-induced depression duration of a recording day (PTDDD) during the delayed neuromonitoring period (PTDDD_delayed_) [7] (Fig. 2). This variable is explained in detail in the COSBID recommendations [59] and denotes the time in minutes in which the most severely affected ECoG channel was in a state of SD-induced depression of spontaneous activity during a recording day. The maximum possible value of PTDDD_delayed_ is 1440 min, as a day has 1440 min. The area under the receiver operating characteristic curve (AUROC) of PTDDD_delayed_ was 0.76 (95% CI, 0.69–0.83; *p* < 0.001) for delayed infarction ipsilateral to the recording strip and 0.88 (95% CI, 0.81–0.94; *p* < 0.001) for DCI (reversible DND or infarction) [7]. For comparison, the AUROC of angiographic vasospasm (digital subtraction angiography (DSA) score of the hemisphere ipsilateral to the subdural electrode strip [7]) was only 0.64 (95% CI, 0.54–0.74; *p* = 0.009) for ipsilateral delayed cerebral infarction and 0.68 (95% CI, 0.54–0.82; *p* = 0.016) for DCI. Peak transcranial Doppler-sonography-(TCD)-determined mean blood flow velocities of the basal cerebral arteries had a similar value for the prediction of delayed infarction and DCI as DSA [7, 10] (Fig. 3b). In multivariate analysis, PTDDD_delayed_ (*β* = 0.474, *p* < 0.001), delayed median Glasgow Coma Score (GCS) (*β* =  − 0.201, *p* = 0.005), and peak TCD-determined mean blood flow velocity of the ipsilateral MCA (*β* = 0.169, *p* = 0.016) explained 35% of the variance in delayed infarction.

In the discussion of Dreier et al. [7], it was suggested how PTDDD_delayed_ may serve to detect reversible neurological deficits and impending infarcts, particularly in unconscious patients, to identify in real-time aSAH patients who are most likely to benefit from targeted management strategies. Thus, a 25-min cut-off for PTDDD_delayed_ seems to be an appropriate first “alert level” to review the patient’s status and initiate tier 1 of targeted management strategies. This recommendation was made although it is still relatively uncertain after 25 min whether the event will be reversible or progress to infarction. It was suggested that tier 1 management adjusts targets for physiological interventions that are expected to modulate the development of SD, such as physiological variables like mean arterial pressure (MAP). If the cumulative SD-induced depression duration of the most recent 24-h period has then exceeded 180 min, the patient has developed a new infarct with 0.62 sensitivity and 0.83 specificity, implying that rescue therapy should not wait 180 min. Therefore, a 60-min cut-off was proposed as an appropriate moment to initiate rescue therapy because it indicates still reversible DND with 0.71 sensitivity and 0.82 specificity. However, ultimately the time delay before starting a therapy is likely to be longer if the intervention has potentially serious side effects than if it has comparatively few side effects. In addition, it was pointed out that automated analysis techniques are under development and will be important in practice if real-time ECoG recordings are used to inform treatment decisions. However, even when such automated analysis techniques are established, it will still be relevant for intensivists to be able to check critical ECoG patterns on the monitor.

Unfortunately, there are a number of pitfalls that can lead to SDs and newly developing infarcts being missed in electrographic recordings. Most of these pitfalls have to do with inadequate recording technique. Figure 3a illustrates one of these pitfalls, namely the loss of information when using subdural recordings that are near-DC, as opposed to full-band DC [22] (a graphical explanation of the technical difference between near-DC and full-band DC recordings can be found in Fig. 1 in Dreier et al. [59]). Attempts to measure SDs with typical intraparenchymal electrode arrays [59, 142–144], epidural electrodes [27], or scalp electrodes [111, 145, 146] are associated with an even greater loss of significance. For example, when intraparenchymal or epidural electrodes are used, around 50% of SDs remain undetected [27, 142]. The DC amplitudes of SDs in epidural recordings are 4 times smaller [27], and in the scalp EEG, they are even 30 times smaller [147] than in the subdural ECoG. In particular, caution is required if signals, e.g., in the scalp EEG, are superficially reminiscent of SDs, but the potential changes are not verified as SDs with parallel subdural ECoG recordings [148]. Since the pattern of true SDs in scalp EEG verified with simultaneous subdural DC/AC-ECoG has already been described [111, 145], it makes sense to first compare one’s own recordings with this pattern before publishing phenomena as SDs that may not be SDs. Overall, electrographic recordings are unfortunately characterized not only by a great abundance of biological events, but also by non-biological artifacts [149].

**Fig. 6:**
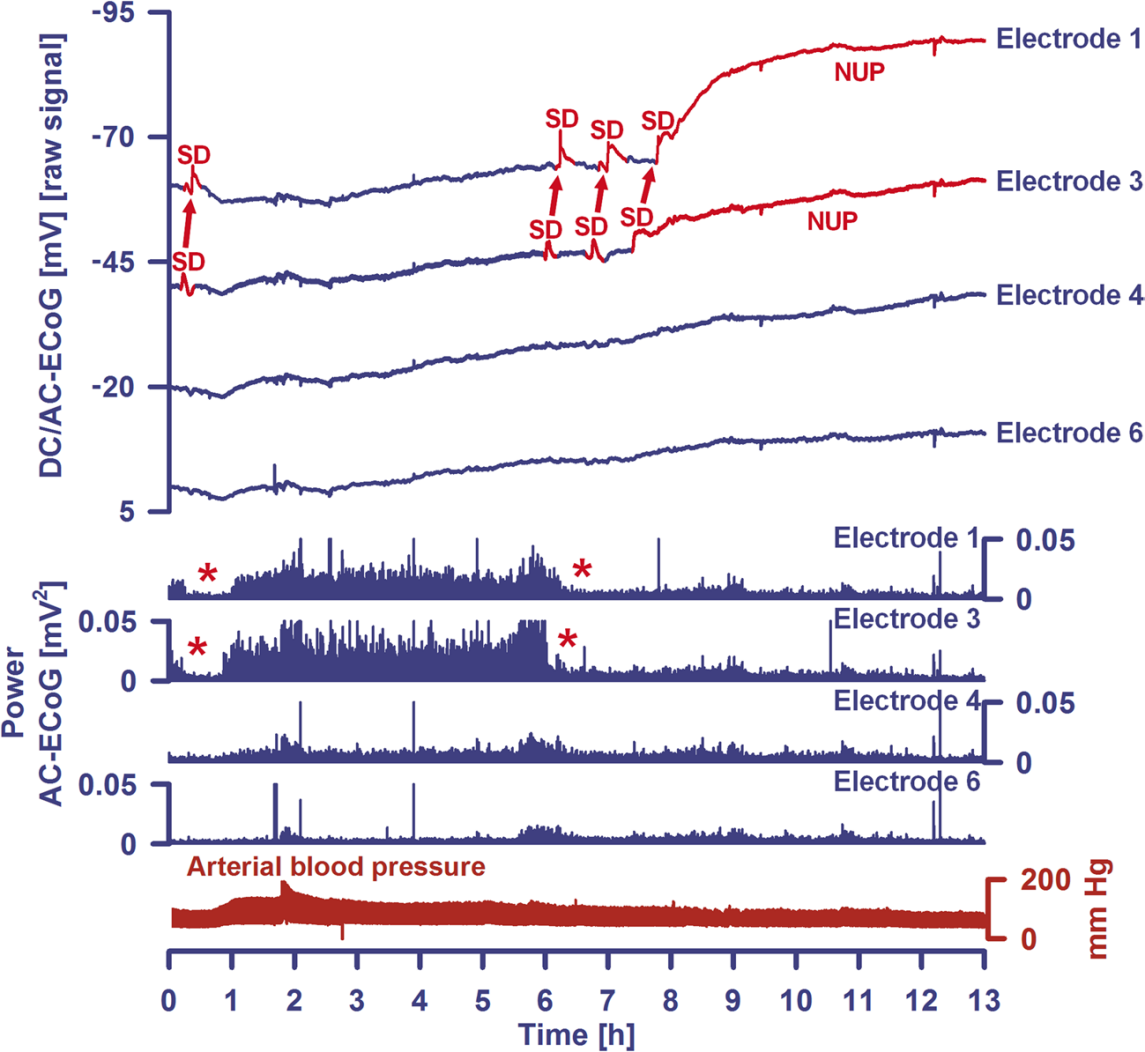
A terminal SD cluster associated with brain death resulting from brainstem compression 36 h before circulatory arrest in a 70-year-old patient. The subdural electrode strip was placed over the viable right frontal cortex. Electrocorticographic (ECoG) recordings only existed from electrodes 1, 3, 4, and 6. The post-interventional MRI on Day 2 showed early cerebral infarcts (10 ml) and a relatively small intracerebral hemorrhage (ICH) (1 ml) ipsilaterally to the recording strip. Before Day 6, the patient had 12 SDs. She also had a relevant early cerebellar infarct (11 ml in the MRI on Day 1). On Day 3, its space-occupying effect required decompressive occipital trepanation [174]. On the same day, she also developed an occlusion of the right femoral artery distal to the insertion site of a Pulse Contour Cardiac Output (PiCCO) device. After an initially successful surgical thrombectomy, the femoral artery subsequently occluded again. Therefore, an attempt of local lysis therapy with rt-PA (0.2 mg/kg) was performed on Day 6. This was unsuccessful. About 2 h later, a cranial CT scan showed a new local hemorrhage at the occipital trepanation site with brainstem compression. Traces 1–4 from top to bottom give the direct current (DC)/alternating current (AC)-electrocorticography (ECoG) recordings (band-pass, 0–45 Hz) at electrodes 1, 3, 4, and 6. The ECoG traces are oriented with negativity upward and positivity downward according to the electroencephalography (EEG) convention. Traces 5–8 show the spontaneous activity in the AC frequency band between 0.5 and 45 Hz. Trace 9 gives the arterial pressure measured with a catheter in the radial artery. Only electrodes 1 and 3 showed spontaneous activity, while the activity had disappeared at electrodes 4 and 6 when the recording restarted approximately 2.5 h after the CT. As can be seen in the figure, an SD occurred at electrode 3 that spread to electrode 1 about 15 min after restart of the recording. The SD led to a spreading depression of activity that lasted for about 50 min (marked by asterisk). This was followed by a stable phase with pronounced delta activity at electrodes 1 and 3 and without further SDs over the next 5 h. Thereafter, the next SD occurred, which also spread from electrode 3 to 1, but the SD-induced spreading depression showed the transition to a persistent depression of spontaneous activity as a sign of imminent deterioration (marked by an asterisk) [59, 110]. The next two SDs followed after about 40 min each. Since these SDs spread in tissue void of spontaneous activity, they meet the criteria of isoelectric SDs [30, 59]. The second of these two SDs then changed into a characteristic negative ultraslow potential (NUP) typical of terminal SD [25]. Thirty-six hours later, terminal extubation resulted in a circulatory arrest. At this point, the DC potential recordings did not reveal any further SD similar to a previous case of brain death [27]. The cause of the delayed SD cluster is speculative. Intracranial pressure (ICP) was not elevated when it began. Nevertheless, upward herniation and local compression is a likely etiology. However, it cannot be completely ruled out that the SDs originated in the brainstem, as a recent model of closed head injury in rats has suggested that SDs can propagate from the cortex to the brainstem [175]. It is conceivable that this is also possible in humans and that it works not only in one direction, but also from the bottom up

Those who want to base clinical decisions on neuromonitoring should use meaningful methods and, as far as SDs are concerned, this is so far only the subdural DC/AC-ECoG (cf. Figures 2, 3, 5, and 6) [59]. With regard to patient safety, linear subdural electrodes, in contrast to electrode grids (which are only used in preoperative epilepsy diagnostics [150]), are not associated with significant complications [7, 29, 32, 128, 151–155] if the recommendations for neurosurgical implantation are followed [156]. In addition, in contrast to intraparenchymal probes [157], linear subdural electrodes do not cause parenchymal injury or local dysfunction of the blood–brain barrier (BBBD), as evidenced with longitudinal MRIs in DISCHARGE-1 [7]. Interesting recent developments include the placement of a slimmer Spencer array subdurally via a burr hole [23, 24] instead of a Wyler electrode strip [22] in patients who do not require a craniotomy. Since an electrode strip can only detect DCI in the hemisphere on which it is placed, this is also a promising way of conducting neuromonitoring of both hemispheres.

## Development of Brain Death During Continued Circulation After aSAH and TBI

**Fig. 7:**
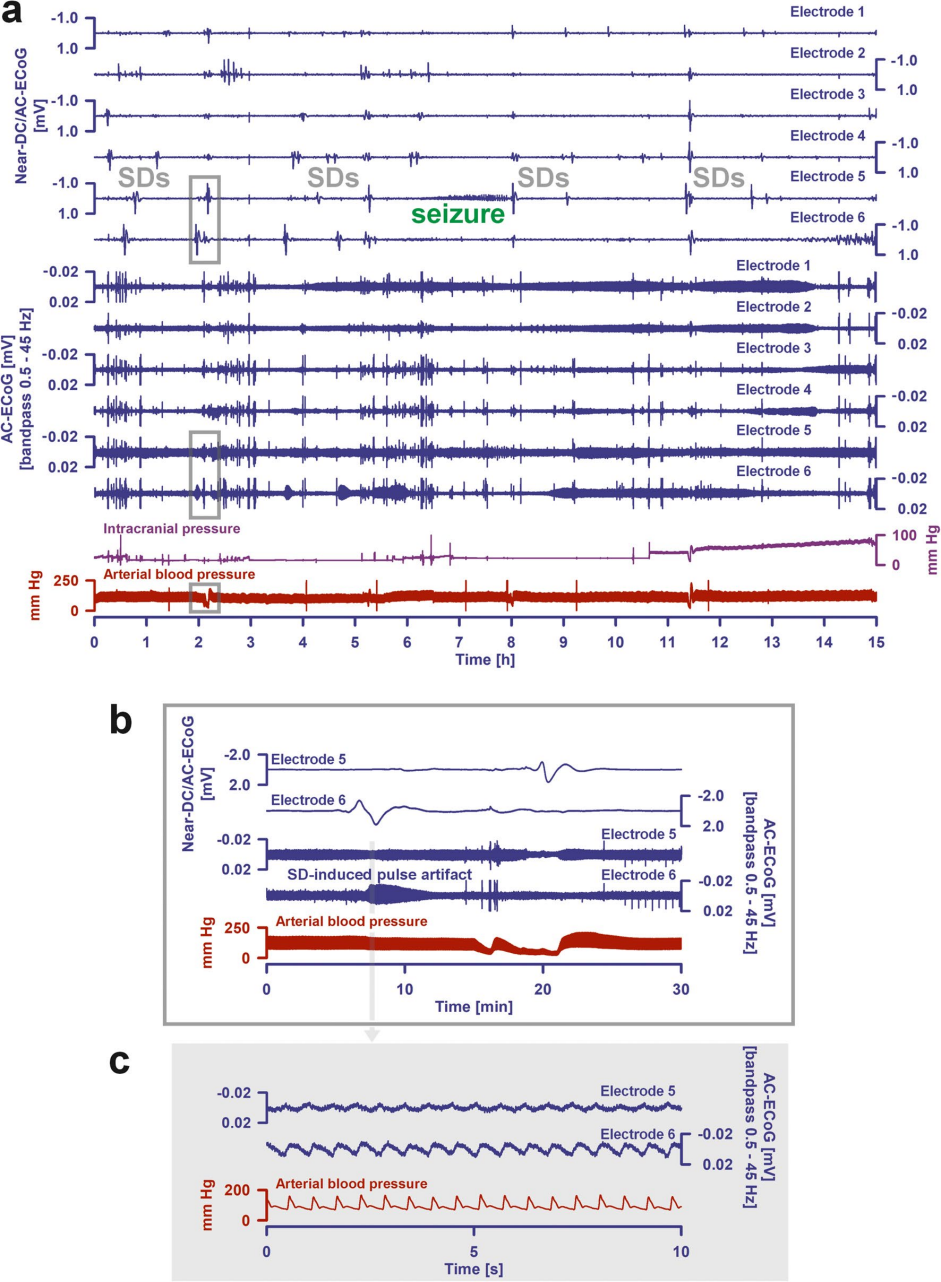
**a** A terminal SD cluster associated with brain death in a 26-year-old patient. The patient suffered bilateral contusions with severe traumatic subarachnoid hemorrhage (tSAH) as a result of a motorcycle accident. Due to the refractory increase in intracranial pressure (ICP) despite maximal conservative therapy, an attempt was made to treat the patient on Day 1 with a bilateral craniectomy [176]. At the end of the surgical procedure, a subdural electrode strip was placed. At the beginning of the subsequent recording phase, the ICP was stable at 20 mmHg. During the following 39 h, an electrographic seizure with typical epileptic spikes of 70-s duration at all electrodes and two single SDs occurred on the background of an otherwise isoelectric electrocorticogram (ECoG) under thiopental anesthesia. At the end of this period, the ICP was stable around 22 mmHg. Then, with gradually increasing frequency, at least 15 SDs occurred, some of which merged into one another. The final 15 h of this cluster of repetitive SDs, during which the ICP rose to 80 mmHg while the mean arterial pressure (MAP) remained at 120 mmHg, is shown in the figure. Traces 1–6 from top to bottom give the near-direct current (DC)/alternating current (AC)-electrocorticography (ECoG) recordings (band-pass, 0.01–45 Hz). The ECoG traces are oriented with negativity upward and positivity downward according to the electroencephalography (EEG) convention. These are monopolar recordings against a platinum-iridium needle electrode placed subcutaneously as a reference on the frontal apex away from muscle attachments. In addition to the multiple SDs, a series of 20–40-s electrographic seizures of increasing amplitude with 50–60-s pauses in between was observed specifically at electrode 5 (marked with “seizure”), which eventually resulted in an SD. Traces 7–12 show irregularly spaced artifacts against a background of ceased spontaneous activity in the AC frequency band between 0.5 and 45 Hz. The ICP can be seen in trace 13. Intermittently, the external ventricular drain (EVD) was left open to counteract the increase in ICP. Accordingly, there are no ICP measurements for these phases. Trace 14 shows the arterial pressure measured with a catheter in the radial artery. One of the SDs is marked with gray boxes and shown in **b** with higher temporal resolution. **b** The SD propagates from electrode 6 to electrode 5. In recordings with an AC amplifier with lower frequency limit of 0.01 Hz, the negative DC shift is distorted but is observed in the near-DC frequency band between 0.01 and 0.05 Hz as a multi-phasic slow potential change that serves to identify SD [59]. Note a typical pulse artifact of isoelectric SDs superimposed on the slow potential change at electrode 6 [82]. This is shown in **c** with higher temporal resolution (gray arrow). Such pulse artifacts are likely caused by SDs due to short-term changes in tissue elasticity superimposed on the increasingly malignant cerebral edema. In detail, they could be related to the regional cerebral blood flow (rCBF) response to SD on the one hand, which may be associated with regional cerebral blood volume changes [22], and to the SD-induced fluid shifts between the intra- and extracellular space [89] and within the glymphatic system [158] on the other. **c** In higher resolution, the pulse artifacts can be identified as pulse artifacts with the help of the arterial pressure curve. Overall, in the context of the patient’s multiple SDs, the presence of severe tSAH in the initial CT was probably of great importance. Thus, Hartings et al. recently showed that of the various anatomic pathologies, only the Morris-Marshall grade of tSAH was significantly associated with the risk of SDs in TBI [29]

The electrographic processes that occur during death are closely related to those that occur during the development of brain infarction. Basically, there are two different biological pathways that lead to the death of the brain: death during cardiocirculatory arrest and brain death during continued systemic circulation. Terminal SD is the electrographic hallmark of both processes. The first study showing terminal SD after cardiocirculatory arrest in patients with aSAH or TBI was published in 2018 [28], closely followed by the first studies showing the characteristic pattern of development of brain death during continued systemic circulation in aSAH patients during neurocritical care [26, 27]. In cardiocirculatory death of patients, as in animals, a simultaneous silencing of neuronal activity (non-spreading depression) develops first, followed by terminal SD with a latency of between 13 and 266 s. In brain death during continued systemic circulation, by contrast, there is a cluster of SDs that can initiate the neuronal silencing in the form of spreading depression, and the SDs eventually transition to a terminal SD characterized by the NUP. The development of ICP increase in the wake of the SD cluster before the development of brain death during continued systemic circulation was then first demonstrated in a DISCHARGE-1 patient [7]. Figures 5 and 7 show two further examples.

Recent experimental evidence suggests that the development of malignant edema with a fatal increase in ICP is at least partly a consequence of SDs [89, 158]. During SD, the concentration gradients of the major ions across the neuronal membranes almost completely collapse (e.g., the intraneuronal calcium concentration increases more than 100-fold and water flows into the neurons (cytotoxic edema)), yet myriads of other small molecules also change their distribution between intra- and extracellular space [67, 75, 90]. Along with the cytotoxic edema caused by SD, an increased content of sodium and chloride ions and water then gradually develops in the tissue if the SD-induced shifts between the intracellular and extracellular space persist [89, 159]. This so-called ionic interstitial edema can be life-threatening because it can increase ICP [89, 160]. Experimentally, cerebrospinal fluid (CSF) surrounding the brain has been identified as the source of ionic edema by penetrating the tissue through perivascular flow channels. This process is initiated by SDs and also by SD-induced spreading ischemia, which further enlarges perivascular spaces and doubles glymphatic inflow speeds [158]. Figure 5 shows a striking example of SD in a patient with aSAH from the DISCHARGE-1 population that triggered spreading ischemia and preceded a terminal crisis of ICP leading to brain death. Figure 6 provides another example of brain death development after aSAH. In this case, a terminal SD cluster resulted from brainstem compression 36 h before circulatory arrest. Figure 7 shows a terminal SD cluster during the development of brain death in a patient with TBI. As expected, the electrographic pattern of brain death development after TBI appears to be very similar to that after aSAH. However, only near-DC/AC-ECoG recordings and no full-band DC/AC-ECoG recordings were available in this patient, which somewhat limits the interpretation, as already explained in Fig. 3a. Of particular interest in Fig. 7 are pulse artifacts in the ECoG of increasingly high amplitude, which seem to occur in association with the SDs, at least in part, and are most likely to be interpreted as an expression of decreased tissue elasticity and increased pulse pressure in the course of brain edema development [64, 82, 161]. SDs were also shown to activate caveolin-1-mediated transport across the BBB [162]. SDs could therefore also contribute to the development of brain edema and ICP increase via BBBD [163]. However, the development of the BBBD-initiated vasogenic interstitial edema is assumed to take several hours longer than the development of the glymphatic system-mediated ionic interstitial edema [89]. Whether these processes can be favorably influenced by drugs that target SDs, such as N-methyl-D-aspartate receptor (NMDAR) antagonists, is currently the subject of intensive preclinical and clinical research [164–170].

## Conclusions

Timely diagnosis of DCI is a serious clinical problem. While physicians can usually recognize acute disturbances of the brain’s energy metabolism in a fully conscious patient through the medical history and neurological examination [59, 171], patients with severe aSAH are often comatose for long periods of time due to the initial injury and sedatives, and the intensivist has little chance of recognizing new neurological deficits against the background of the pre-existing global neurological deficit. Therefore, diagnosis of DCI is usually delayed in the critical care setting, and rescue treatment cannot be provided at the appropriate time. In DISCHARGE-1, for example, 90/170 of the early survivors (52.9%) were clinically unassessable during the entire observation period of 2 weeks. To make matters worse, these comatose patients also had a significantly larger delayed infarct volume than patients for whom clinical assessment was at least partially feasible [7].

In this regard, regional ECoG monitoring offers a significant advantage since it allows local and even remote detection of developing DCI. This is due to the fact that SDs propagate widely from metabolically stressed zones and characteristic patterns, including temporal clusters of SDs and persistent depression of spontaneous brain activity, can be recognized and quantified. The spread of SDs and the widespread changes in spontaneous activity that they entail is a particular advantage of ECoG over other neuromonitoring modalities, such as microdialysis or brain tissue partial pressure of oxygen measurements, which record only local conditions and may not detect clinically important changes that develop elsewhere in an injured lobe or hemisphere [10].

Other techniques for predicting DCI are, for example, perfusion CT or perfusion MRI. Positron emission tomography (PET), unlike perfusion CT and perfusion MRI, even allows absolute measurements of rCBF. However, none of these techniques allows continuous measurements in real time, and concerning PET, the most informative among these imaging modalities, Minhas et al. [136] concluded the following: “A markedly heterogeneous pattern of CBF distribution was observed, with hyperemia, normal CBF values, and reduced flow being observed among patients with delayed neurological deficits… Among patients who developed delayed neurological deficits after aneurysmal subarachnoid hemorrhage, a wide range of cerebral perfusion disturbances was observed, calling into question the traditional concept of large-vessel vasospasm.” These PET observations fit perfectly with the invasively measured neuromonitoring data, in which the course of rCBF in patients with DCI was highly dynamic. Thus, in SD-induced spreading ischemia, rCBF typically falls locally from sometimes quite high to ischemic values within seconds and may remain in the ischemic range for up to 2.5 h [22]. After the ischemic phase, rCBF may rise to extremely high levels, well above the initial baseline, to gradually return to normal or somewhat reduced levels over several tens of minutes until the next SD-induced spreading ischemia occurs (for example, compare Fig. 7 in [22]) or Fig. 6 in [25]). Accordingly, the neuromonitoring methods discussed here have enormous potential, as they map these highly dynamic changes in real time and with greater accuracy than previous methods. Only the development of adequate diagnostic procedures for the timely detection of patients in need of therapy offers the chance to identify patients who could benefit from a given treatment and selectively treat them at the right time; meanwhile, the remaining patients without such complications could be spared the side effects of unnecessary interventions. It would therefore be a fallacy to use the current lack of evidence-based rescue therapies for DCI [20, 172] as an argument for not developing precision medicine for DCI. The lack of such therapies is only a logical consequence of the lack of appropriate diagnostic methods. The advances in neuromonitoring and understanding of brain lesion development, as outlined here, offer hope that precision medicine for aSAH and similar acute neurologic diseases can still be achieved.

## Data Availability

Electronic recording, processing and storage of the data were approved by the data protection officer of the Charité – Universitätsmedizin Berlin (data protection votes from 28 May 2008 and 5 May 2014). The datasets analyzed are not publicly available because the patient’s informed consent only permits the data analysis and publication by the investigators.

## Abbreviations

AC: Alternating current
aSAH: Aneurysmal subarachnoid hemorrhage
AUROC: Area under the receiver operating characteristic curve
BBB: Blood-brain barrier
BBBD: Blood-brain barrier dysfunction
CBF: Cerebral blood flow
95% CI: 95% Confidence interval
COSBID: Co-Operative Studies on Brain Injury Depolarizations
CT: Computed tomography
DALY: Disability-adjusted life years
DC: Direct current
DCI: Delayed cerebral ischemia
DISCHARGE-1: Depolarizations in ischemia after subarachnoid hemorrhage-1
DND: Delayed neurological deficit
DSA: Digital subtraction angiography
ECI: Early cerebral ischemia
ECoG: Electrocorticography
GCS: Glasgow Coma Score
ICH: Intracerebral hemorrhage
ICP: Intracranial pressure
IQR: Interquartile range
MAP: Mean arterial blood pressure
MCA: Middle cerebral artery
MCAo: Middle cerebral artery occlusion
MRI: Magnetic resonance imaging
NMDAR: N-methyl-D-aspartate receptor
NOS: Nitric oxide synthase
NUP: Negative ultraslow potential
PET: Positron emission tomography
PTDDD: Peak total spreading depolarization-induced depression duration of a recording day
PTDDD_delayed_: Peak total spreading depolarization-induced depression duration of a recording day in the delayed neuromonitoring period
SAH: Subarachnoid hemorrhage
SD: Spreading depolarization
TBI: Traumatic brain injury
TCD: Transcranial Doppler-sonography
TIA: Transient ischemic attack
tSAH: Traumatic subarachnoid hemorrhage
YPLL: Proportion of potential years of life lost before age 65
